# Short-term smoking cessation leads to a universal decrease in whole blood genomic DNA methylation in patients with a smoking history

**DOI:** 10.1186/s12957-023-03099-2

**Published:** 2023-07-26

**Authors:** Junyi Shang, Xinran Nie, Yanan Qi, Jing Zhou, Yong Qi

**Affiliations:** 1 Department of Respiratory and Critical Care Medicine, Henan Provincial People’s Hospital, People’s Hospital of Zhengzhou University; People’s Hospital of Henan University, No. 7 Weiwu Road, Jinshui District, Zhengzhou, 450003 Henan China; 2 Academy of Medical Science, Zhengzhou University, Zhengzhou, China; 3 Department of Respiratory and Critical Care Medicine, People’s Hospital of Zhengzhou University, Zhengzhou, 450003 Henan China; 4 Department of Respiratory and Critical Care Medicine, Central China Fuwai Hospital; Central China Fuwai Hospital of Zhengzhou University; People’s Hospital of Zhengzhou University; Henan Provincial People’s Hospital, Zhengzhou, 450003 Henan China; 5 Department of Health Management, Henan Provincial People’s Hospital, Henan University People’s Hospital, Zhengzhou, 450003 China

**Keywords:** Epigenetics, Smoking cessation, DNA methylation, Cell cycle, Pan-cancer

## Abstract

**Background:**

Epigenetics is involved in various human diseases. Smoking is one of the most common environmental factors causing epigenetic changes. The DNA methylation changes and mechanisms after quitting smoking have yet to be defined. The present study examined the changes in DNA methylation levels before and after short-term smoking cessation and explored the potential mechanism.

**Methods:**

Whole blood and clinical data were collected from 8 patients before and after short-term smoking cessation, DNA methylation was assessed, and differentially methylated sites were analyzed, followed by a comprehensive analysis of the differentially methylated sites with clinical data. GO/KEGG enrichment and protein–protein interaction (PPI) network analyses identified the hub genes. The differentially methylated sites between former and current smokers in GSE50660 from the GEO database were detected by GEO2R. Then, a Venn analysis was carried out using the differentially methylated sites. GO/KEGG enrichment analysis was performed on the genes corresponding to the common DNA methylation sites, the PPI network was constructed, and hub genes were predicted. The enriched genes associated with the cell cycle were selected, and the pan-cancer gene expression and clinical significance in lung cancer were analyzed based on the TCGA database.

**Results:**

Most genes showed decreased DNA methylation levels after short-term smoking cessation; 694 upregulated methylation CpG sites and 3184 downregulated methylation CpG sites were identified. The DNA methylation levels were altered according to the clinical data (body weight, expiratory, and tobacco dependence score). Enrichment analysis, construction of the PPI network, and pan-cancer analysis suggested that smoking cessation may affect various biological processes.

**Conclusions:**

Smoking cessation leads to epigenetic changes, mainly decreased in the decline of most DNA methylation levels. Bioinformatics further identified the biologically relevant changes after short-term smoking cessation.

## Background

Smoking is a preventable risk factor for many diseases, including respiratory illness, cardiovascular diseases, and cancers. Tobacco inhalation is one of the leading risk factors for lung cancer [1]. Heavy smokers’ cumulative lung cancer risk may be approximately 30% higher than never smokers [2]. The effect of smoking on cardiovascular disease cannot be ignored in adults or children [3]. The roles of smoking in disease may involve multiple molecular and cellular mechanisms; however, the specific mechanisms have not yet been investigated [4, 5].

Epigenetics refer to heritable changes that alter gene expression without modifying the DNA sequence. Many studies have focused on the impact of environmental and lifestyle factors on abnormal epigenetic status and disease, with smoking habits receiving significant attention [6, 7]. DNA methylation, with the addition of a methyl group at the 5’ position of cytosine in CpG dinucleotides, indicates an epigenetic modification, which regulates gene expression and protects genome integrity. Epigenetic changes are the hallmark of cancer [8] and are associated with obesity, diabetes mellitus, and others [9, 10]. Cigarette smoke is one of the most potent environmental modulators of DNA methylation, and several genome-wide association studies have identified the differentially methylated CpG sites (DMCpGs) related to smoking [11]. These sites are associated with gene expression and may exert several roles in cellular, hematological, immune, cardiovascular, carcinogenic, and other mechanisms and functions [12, 13].

The earlier smokers successfully quit smoking, the lower the mortality rate [14]. Regardless of age, quitting smoking seems to reduce mortality at all ages [15]. DNA methylation is a reversible process for smokers, and some smoking-induced differentially methylated CpG sites partially return to a never-smoker state due to smoking cessation [16–18]. The most relevant article described “long-term smoking cessation” as > or = 6 months [19, 20]. Although some studies focus on long-term smoking cessation, few have assessed short-term cessation interventions.

In the present study, DNA methylation sequencing before and after short-term smoking cessation was performed, and differentially methylated CpG sites and the corresponding genes were identified, then, assessed the correlation between changes in methylation and clinical features. Finally, bioinformatics analysis was carried out to further explore the functions of these genes.

## Methods

### Inclusion criteria

All human studies were approved by the Henan Provincial People’s Hospital ethics committee. According to the Chinese Guidelines for Smoking Cessation (2015 Edition), smoking cessation intervention was performed with varenicline tartrate for smokers. Primary screening was conducted on smokers who visited the smoking cessation clinic of Henan Provincial People’s Hospital from August 2018 to December 2019. Pulmonary function tests were conducted according to the patient’s wishes or the clinician’s evaluation. Whole blood and clinical data were collected separately before (pre-quitting group) and 3–6 months after smoking cessation intervention (post-quitting group). After the screening, 8 males were enrolled in this study.

### DNA methylation assay

Whole blood samples from 8 subjects before and after smoking cessation intervention were collected to extract genomic DNA. The following DNA methylation analysis processes followed the Illumina Human 850 K BeadChip instructions [21]. Genomic DNA was quantified on a spectrophotometer (CapitalBio MedLab, Beijing, China), adjusted to standard solution concentrations of 50 ng/μL and 20 μL, and then detected by 0.8% agarose gel electrophoresis as a 10-kbp band without any degradation. A total amount of more than 5 μg can be used in chromatin immunoprecipitation (ChIP) experiments. Sulfite conversion was carried out according to the optimization method of the Zymo EZ DNA Methylation kit officially recommended by Illumina. Then 0.1 N NaOH was added to the sample to denature the DNA into single strands. After neutralization, the whole genome amplification reagent was added and incubated overnight at 37 °C. The amplified product was subjected to enzymatic hydrolysis to obtain fragmented DNA. Isopropanol was added, and DNA fragments were centrifuged at 4 °C. After the precipitated DNA was air-dried, a hybridization buffer (Illumina) was added to redissolve the precipitated DNA. The resuspended DNA sample was hybridized overnight with the prepared chip. The fragmented DNA was denatured and connected with 50 bases at the specific site in hybridization. All unhybridized or mismatched DNAs were removed. Dinitrophenol and biotin-labeled nucleotide substrates (A/T and C/G) in the extension base of the capture probe and only the probe with complementary binding to gDNA could be extended. Different fluorescent dyes were labeled A/T and C/G by staining. The corresponding manifest file was downloaded, ChIP-seq was fitted into the scanner, the raw data were generated, the raw data were imported into R-package ChAMP software for analysis, and finally, the methylation level of each site for each sample was obtained.

### Identification of differentially methylated CpG sites and differentially methylated regions

Raw data were loaded into the R package ChAMP in the form of IDAT files. Individual CpG sites were obtained for each patient sample, quality control of the raw data was implemented, and normalization was carried out via BMIQ. Differential methylation analysis was performed using R Limma3.32.10; when the *P* value was < 0.001, samples were divided into up- or downregulated methylation groups based on beta values of < 0 or > 0, respectively. Volcano plots were plotted for the methylated sites using R software (version 3.6.3) and the R package survival (version 3.3.3). The data were plotted as pie charts according to the distribution of the CpG sites in CpG islands that were further divided into CpG island/shore/shelf/open sea. A Circos diagram was generated to display the screened significantly different CpG site distribution over the genome, and clustering analysis was performed using Cluster 3.0. Gene Ontology (GO)/Kyoto Encyclopedia of Genes and Genomes (KEGG), protein–protein interaction (PPI) analysis and identification of hub genes were based on the genes corresponding to up- or downregulated methylation, respectively. GO/KEGG analyses were carried out using R (version 3.6.3) and the clusterProfiler package (version 3.14.3); PPI networks were constructed using STRING, and hub genes were identified via the CytoHubba plugin in Cytoscape (version 3.7.1).

### Correlation between differential methylation and clinical data

Spearman’s correlation analysis identified the differential methylation from the paired data before and after smoking cessation, with screening conditions of the absolute value of the correlation coefficient > 0.8 and *P* value < 0.05. The results are expressed in heatmaps. R (version 3.6.3) and the R package ggplot2 (version 3.3.3) were utilized for statistical analyses and visualization.

### Identification of the same differentially methylated CpG sites in with GSE50660

The NCBI GEO database (https://www.ncbi.nlm.nih.gov/geo/) was searched using “cigarette smoking” and “DNA methylation” as search terms. After the screening, GSE50660 was identified for subsequent analysis. GSE50660 used Illumina Infinium HumanMethylation450 BeadChip to measure the DNA methylation levels of the current, former, and never-smokers, while GEO2R was used for differentially expressed DNA methylation between former and current smokers. The screening of differentially methylated CpGs was carried out with a threshold of *P* value < 0.05. A Venn diagram was constructed to display these differentially methylated CpG sites of GSE50660 and the downregulated methylated CpG sites of the data from these 8 patients using the ggplot2 packages (version 3.3.3). GO/KEGG analysis, PPI analysis, and hub gene prediction were performed on the genes corresponding to the methylation sites obtained by the Venn diagram. Cell cycle abnormalities are a driving force in tumorigenesis. The genes related to the cell cycle obtained by cluster analysis were analyzed in the TCGA pan-cancer cohort. The RNAseq data were downloaded from UCSC XENA (https://xenabrowser.net/datapages/). The transcripts per million reads (TPM) format data was analyzed after log2 conversion. Statistical analyses and mapping were performed using R (version 3.6.3) and ggplot2 packages (version 3.3.3). The TCGA-LUAD and TCGA-LUSC RNAseq data were downloaded from the TCGA database (https://portal.gdc.cancer.gov). Statistical analyses and mapping were performed using R (version 3.6.3), stats(version 4.2.1), car (version 3.1–0), pROC (version 1.18.0), and ggplot2 packages (version 3.3.3).

## Results

### Altered DNA methylation levels before and after short-term smoking cessation

**Fig. 1:**
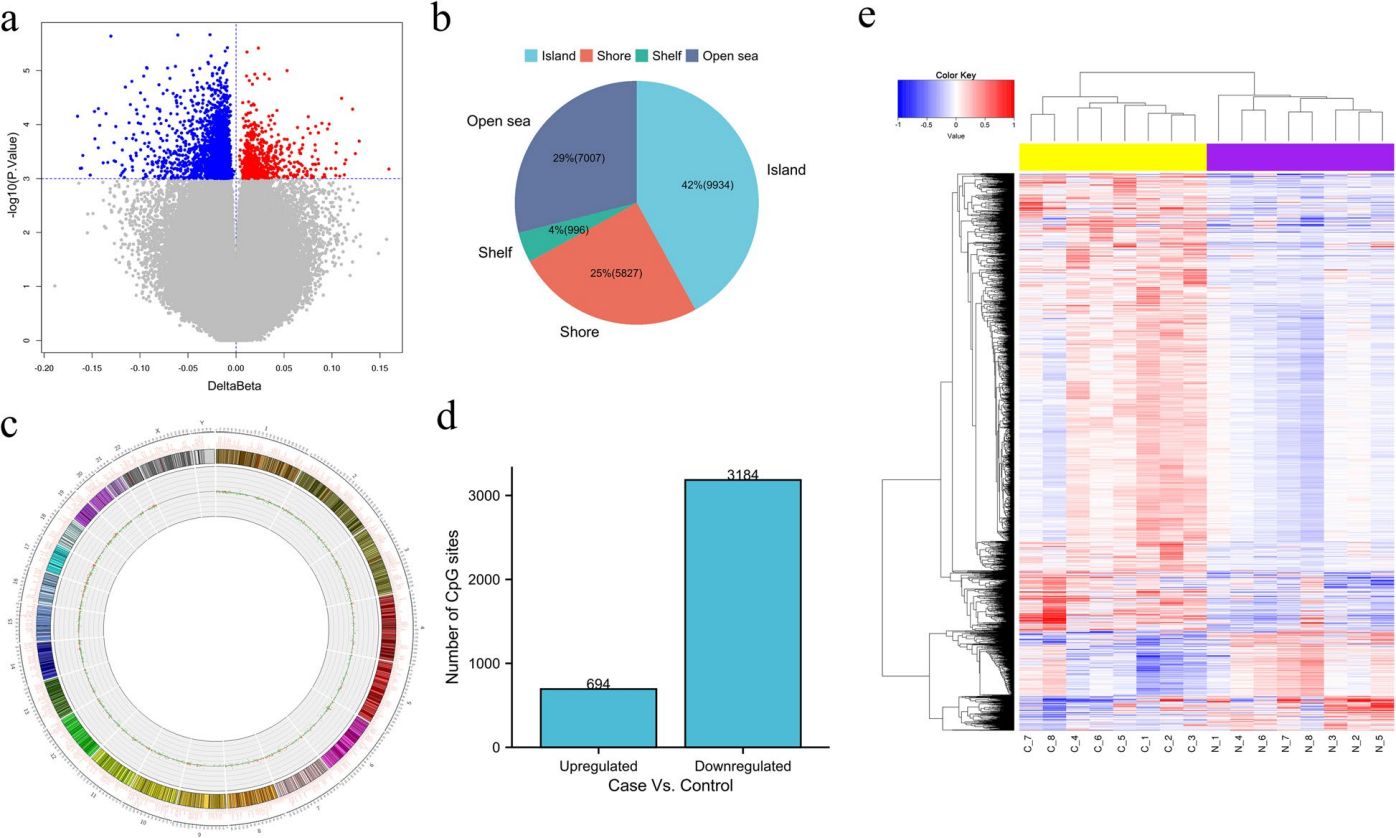
Distribution of the differentially methylated CpG sites and clustering analysis before and after short-term smoking cessation. **a** Volcano plot of differentially methylated sites: red represents methylation levels elevated after short-term smoking cessation, blue represents methylation levels that declined after short-term smoking cessation. **b** Distribution of the CpG sites in CpG categories. **c** Genome-wide distribution of the differentially CpG sites. **d** The CpG sites number of short-term smoking cessation(Case) compared with before short-term smoking cessation (Control). **e** Graph-clustering was used to display these differentially methylated CpG sites and clustering analysis of their methylation values. Samples are accommodated in columns and DMCpGs in rows in the matrix. Colorbar denotes a comparison table of number and color (relatively high in red and relatively low in blue), the top sample tree represents a similar degree clustering relationship between samples, and samples of the same color indicate the same group. C1-C8: Control. N1-N8: Case. The darker the color, the greater the MCC value

Paired analysis of the methylation levels of 8 patients before and after short-term smoking cessation showed differential expression in 3878 CpG sites (*P* < 0.001) (Fig. 1a). Regarding CpG distribution, islands account for 42%, open seas account for 29%, shelves account for 4%, and shores account for 25% of CpG sites (Fig. 1b). A Circos plot was constructed for the whole genome display (Fig. 1c). 694/3878 differential methylation CpG sites were upregulated and 3184/3878 downregulated (Fig. 1d). Finally, a cluster graph was constructed to display these differentially methylated CpG sites and analyze their methylation values (Fig. 1e).

### Correlation between differentially methylated sites and clinical features before and after short-term smoking cessation

**Fig. 2:**
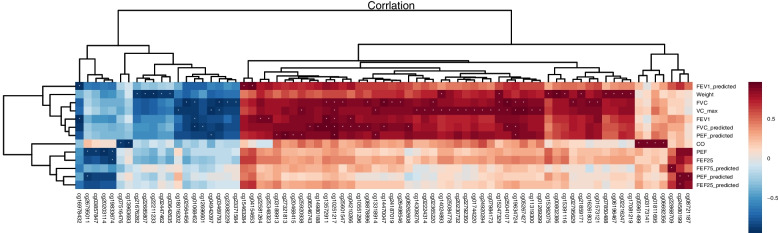
The correlation analysis between differentially methylated CpG sites and clinical information. The abscissa represents different methylated CpG sites, the ordinate represents the clinical information differently. Red colors represent a positive correlation and blue colors represent a negative correlation, the darker the color, the larger the correlation values

The clinical data, including body weight, tobacco dependence score, and lung function, were collected from 8 patients before and after short-term smoking cessation. The tobacco dependence score was based on the Fagerstrom test for nicotine dependence (FTND), and data were analyzed using a paired-sample *t*-test (Table 1). A correlation analysis was performed between differentially methylated CpG sites and clinical features, and significant correlations were indicated by absolute values of the correlation coefficients > 0.8 and *P* value < 0.05 (Fig. 2).

**Table 1:** Clinical data of subjects before and after short-term smoking cession

	Before	After	*P* value
Body weight (kg)	77.23 ± 10.24	79.62 ± 12.48	0.026*
Expiratory CO (ppm)	8.15 ± 2.97	3.01 ± 1.04	0.000**
Tobacco dependence score	6.69 ± 2.02	0.00 ± 0.00	0.000**
FEV1 (L)	2.80 ± 0.80	2.84 ± 0.79	0.337
FEV1% pred	83.21 ± 20.87	85.02 ± 19.16	0.459
FVC (L)	3.84 ± 0.00	3.97 ± 0.87	0.277
FVC% pred	96.09 ± 21.56	100.47 ± 17.39	0.133
FEV1/FVC%	72.89 ± 10.23	70.79 ± 8.66	0.301
FEF25 (L/S)	4.18 ± 2.03	5.02 ± 1.92	0.043*
FEF25% pred	53.26 ± 21.78	62.21 ± 23.08	0.076
FEF50 (L/S)	3.17 ± 1.63	3.06 ± 1.56	0.623
FEF50% pred	68.01 ± 36.89	65.81 ± 35.77	0.632
FEF75 (L/S)	1.22 ± 0.96	0.90 ± 0.44	0.321
FEF75% pred	51.66 ± 17.74	47.30 ± 19.92	0.338
MMEF (L/S)	2.30 ± 0.99	2.21 ± 1.01	0.599
MMEF% pred	59.44 ± 25.65	56.76 ± 24.49	0.550
PEF (L/S)	5.13 ± 1.83	5.93 ± 1.66	0.054
PEF% pred	53.63 ± 18.41	61.58 ± 16.61	0.096

^*^P < 0.05; **P < 0.001

### GO/KEGG analysis, PPI network construction, and hub gene identification based on the genes corresponding to upregulated methylation

**Fig. 3:**
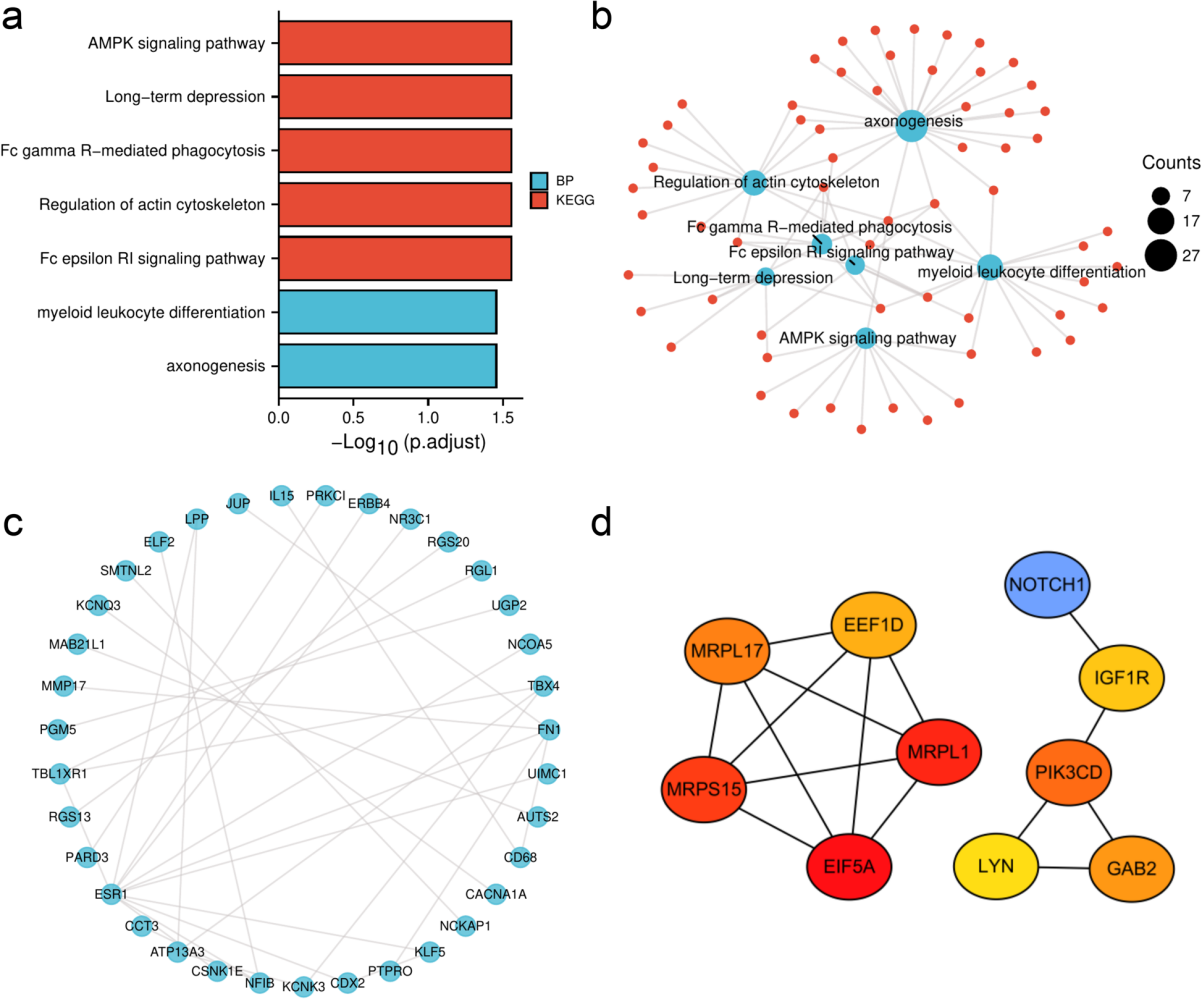
The analysis of genes corresponding to the methylation sites up-regulated. **a** Histograms of GO/KEGG analysis, blue represent BP of GO analysis, red represent KEGG analysis. **b** Divergence diagram of GO/KEGG analysis, the size of the circle represents the number of enrichment. **c** PPI network of the top 100 genes corresponding to the methylation sites up-regulated. **d** The top 10 hub genes

Enrichment analysis examined the biological relevance of genes corresponding to the upregulated methylation sites. The GO analysis results showed that the majority of enriched categories were myeloid leukocyte differentiation and axonogenesis. The KEGG analysis showed that the most enriched categories were the AMPK signaling pathway, long-term depression, Fc gamma R-mediated phagocytosis, regulation of actin cytoskeleton, and Fc epsilon RI signaling pathway. The GO/KEGG analysis results are displayed in the histogram (Fig. 3a) and divergence diagram simultaneously (Fig. 3b).

The top 100 genes corresponding to the upregulated methylation sites were entered into the STRING database (https://cn.string-db.org/) to obtain PPIs; the results were visualized using the Igraph package (version 1.2.6) (Fig. 3c). The top 10 hub genes were acquired from Cytoscape using the MCC algorithm (Fig. 3d).

### GO/KEGG analysis, PPI network construction, and hub genes identified based on the genes corresponding to downregulated methylation

**Fig. 4:**
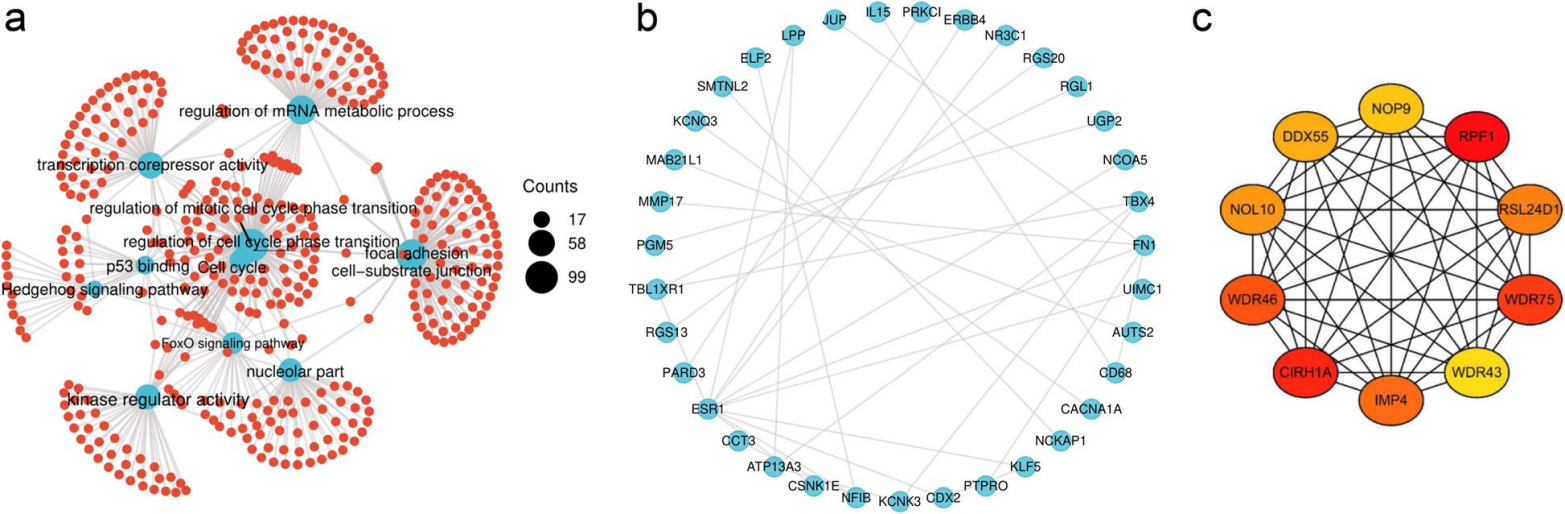
The analysis of genes corresponding to the methylation sites down-regulated. **a** Divergence diagram of GO/KEGG analysis, the size of the circle represents the number of enrichment. **b** PPI network of the top 100 genes corresponding to the methylation sites down-regulated. **c** The top 10 hub genes

The main enriched terms in the GO analysis were kinase regulator activity, P53 binding, transcription corepressor activity, cell-substrate junction, focal adhesion, nucleolar part, regulation of mitotic cell cycle phase transition, cell cycle phase transition, and mRNA metabolism. The main enriched pathways in the KEGG analysis were the Hedgehog signaling pathway, the FOXO signaling pathway, and the cell cycle (Fig. 4a). The top 100 genes corresponding to the downregulated methylation sites were obtained from the STRING platform (Fig. 4b). The hub genes based on the genes corresponding to the downregulated methylation sites are shown in Fig. 4c.

### Differentially methylated CpGs with GSE50660

**Fig. 5:**
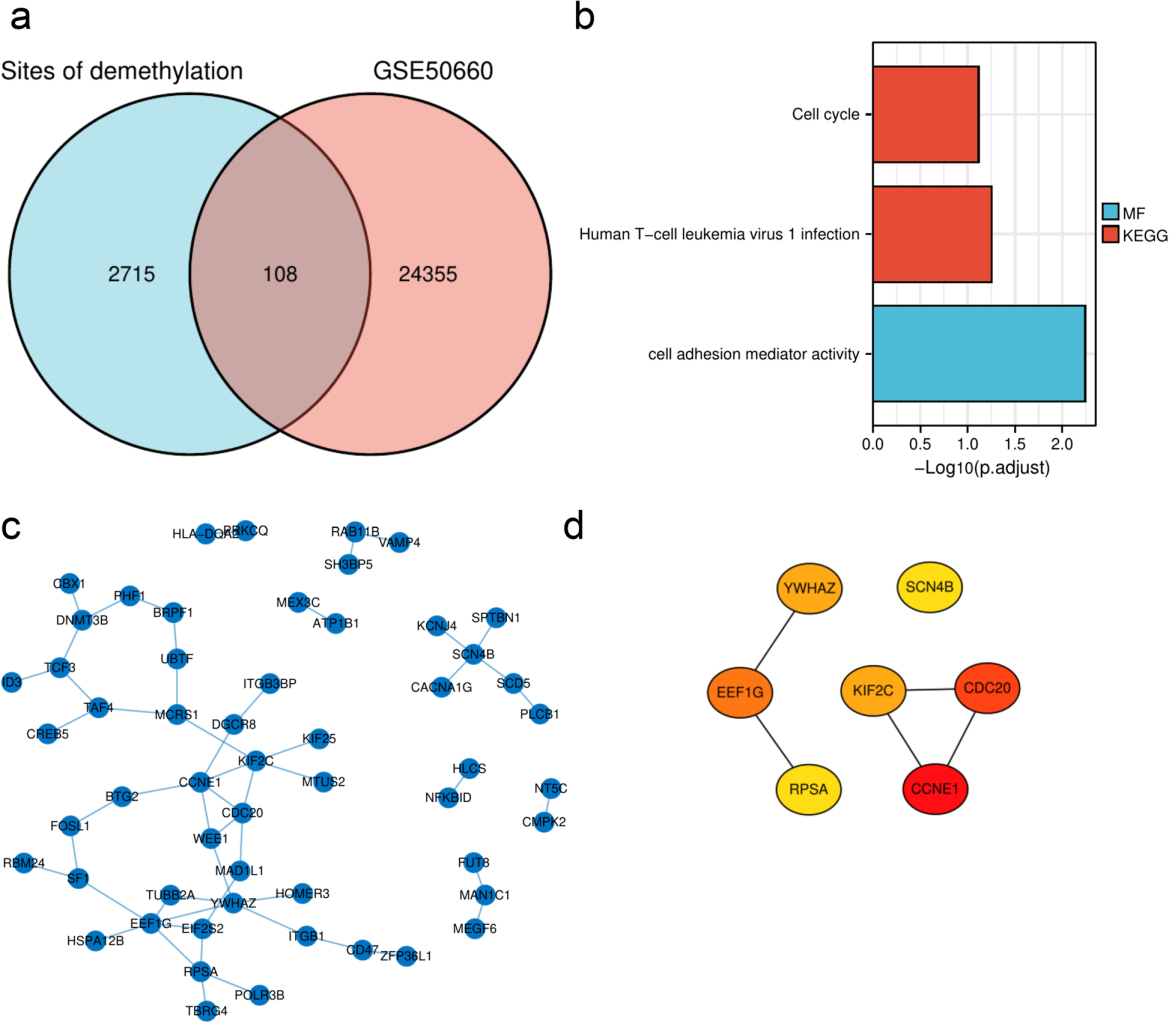
The analysis between the 8 patients’ data and GSE50660. **a** The Venn diagram is based on the following data: differentially methylated CpG sites of GSE50660 between former and current smokers, and the down-regulated methylation sites of the 8 patients’ data. **b** Histograms of GO/KEGG analysis, blue represents MF of GO analysis, and red represents KEGG analysis. **c** PPI network of the genes corresponding to these 108 methylated CpG sites. **d** The hub genes correspond to these 108 methylated CpG sites

The Venn diagram showed that 108 methylated CpG sites overlapped between the data of these 8 patients and GSE50660 (Fig. 5a). The GO/KEGG analysis for the genes corresponding to these 108 methylated CpG sites is shown in Fig. 5b. A PPI network was constructed for the genes corresponding to these 108 methylated CpG sites (Fig. 5c) and the top 7 hub genes (Fig. 5d) were identified.

### Pan-cancer analysis

**Fig. 6:**
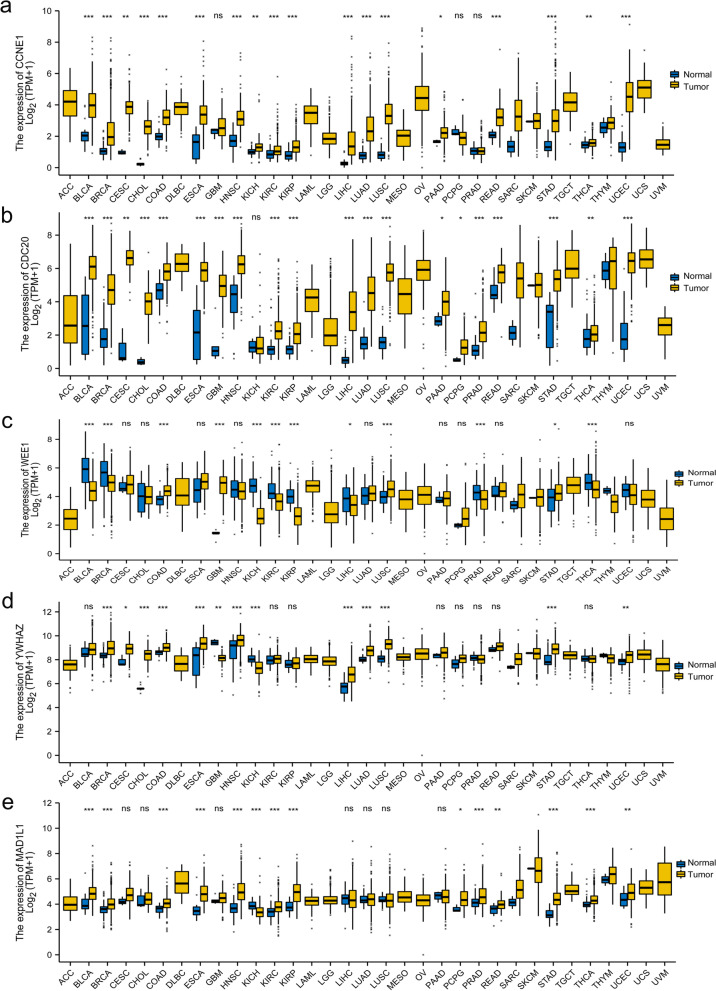
The five genes associated with the cell cycle in pan-cancer using unpaired analysis. The abscissa represents different tumor types; the ordinate represents gene expression values. **a** The expression of CCNE1 in pan-cancer. **b** The expression of CDC20 in pan-cancer. **c** The expression of WEE1 in pan-cancer. **d** The expression of YWHAZ in pan-cancer. **e** The expression of MAD1L1 in pan-cancer

**Fig. 7:**
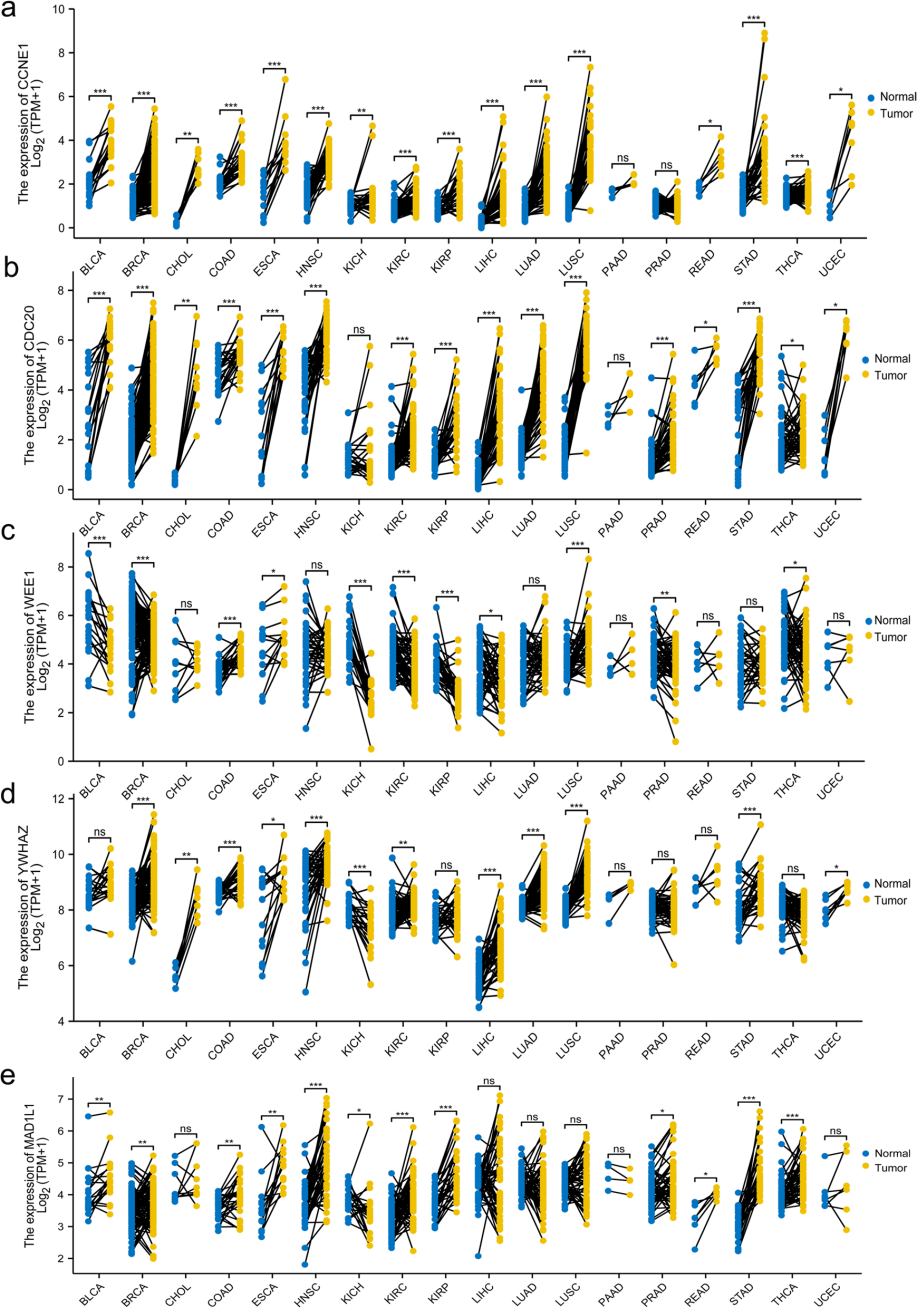
The five genes associated with the cell cycle in pan-cancer using paired analysis. **a** The expression of CCNE1 in pan-cancer. **b** The expression of CDC20 in pan-cancer. **c** The expression of WEE1 in pan-cancer. **d** The expression of YWHAZ in pan-cancer. **e** The expression of MAD1L1 in pan-cancer

**Fig. 8:**
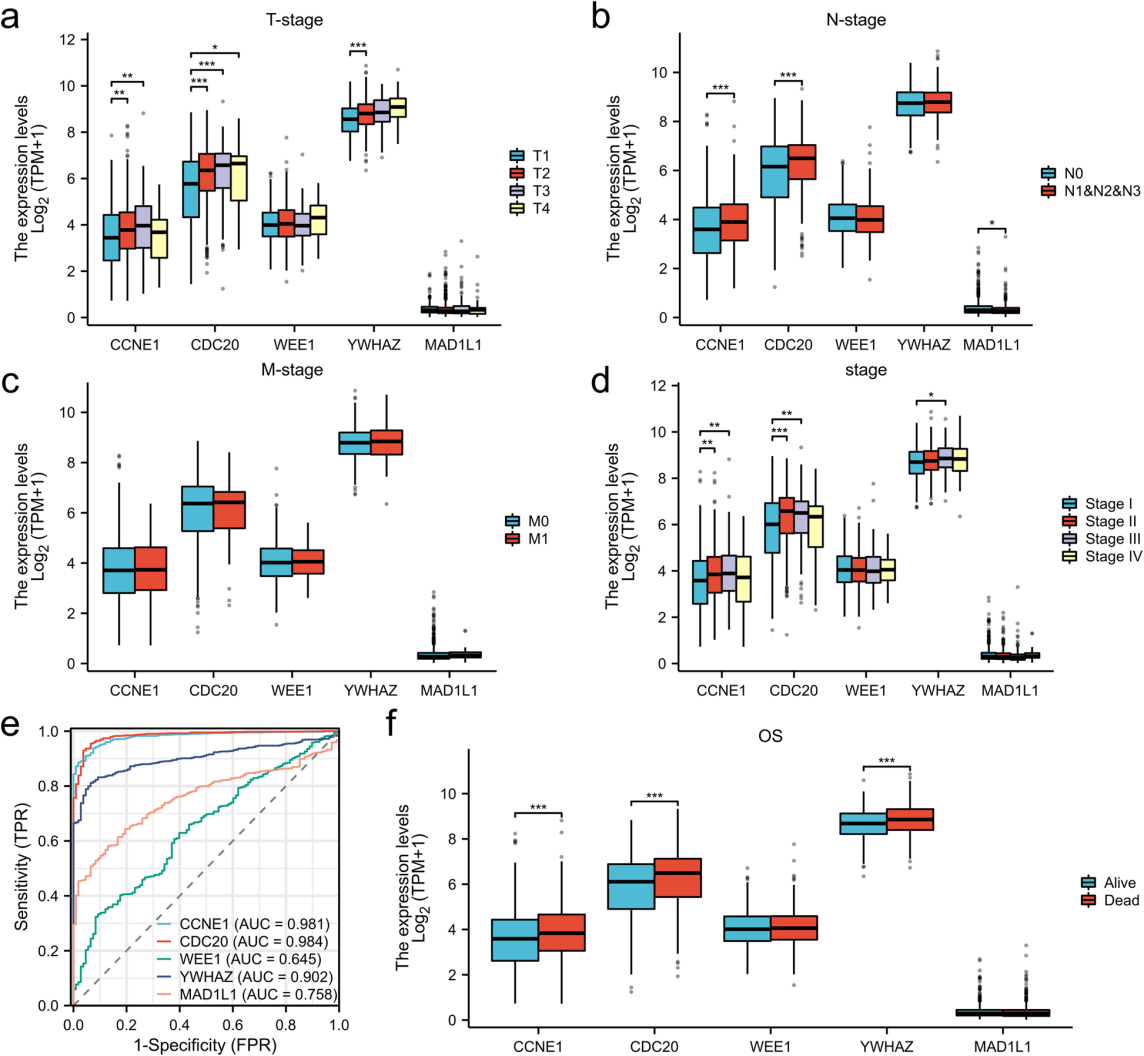
The clinical outcomes of CCNE1, CDC20, WEE1, YWHAZ, and MAD1L1 in lung cancer. **a** The gene expression for T-stage. **b** The gene expression for N-stage. **c** The gene expression for M-stage. **d** The gene expression for the pathologic stage. **e** The ROC curves of CCNE1, CDC20, WEE1, YWHAZ, and MAD1L1 to lung cancer. **f** The gene expression on overall survival (OS) in lung cancer

In the KEGG analysis (Fig. 5), five genes were related to the cell cycle (Table 2). The expression of these five genes in the pan-cancer dataset was assessed using unpaired (Fig. 6) or paired analysis (Fig. 7). The clinical significance of these five genes in lung cancer is shown in Fig. 8. Table 3 presents the abbreviations of the 33 types of cancer.

**Table 2:** The gene list of GO/KEGG analysis for the genes corresponding to 108 genes in common

Ontology	ID	Description	Gene ID
MF	GO: 0098631	Cell adhesion mediator activity	CD47 / ITGB1 / RPSA / STXBP6 / VSTM2L
KEGG	hsa05166	Human T-cell leukemia virus 1 infection	CCNE1 / CDC20 / HLA-DQA2 / TCF3 / FOSL1 / MAD1L1 / CREB5
KEGG	hsa04110	Cell cycle	CCNE1 / CDC20 / WEE1 / YWHAZ / MAD1L1

**Table 3:** The abbreviation of 33 types of cancer

Full name	Abbreviation	Full name	Abbreviation	Full name	Abbreviation
Adrenocortical carcinoma	ACC	Bladder Urothelial Carcinoma	BLCA	Breast invasive carcinoma	BRCA
Cervical squamous cell carcinoma and endocervical adenocarcinoma	CESC	Cholangiocarcinoma	CHOL	Colon adenocarcinoma	COAD
Lymphoid neoplasm diffuse large B-cell lymphoma	DLBC	Esophageal carcinoma	ESCA	Glioblastoma multiforme	GBM
Head and neck squamous cell carcinoma	HNSC	Kidney chromophobe	KICH	Kidney renal clear cell carcinoma	KIRC
Kidney renal papillary cell carcinoma	KIRP	Acute myeloid leukemia	LAML	Lower grade glioma	LGG
Liver hepatocellular carcinoma	LIHC	Lung adenocarcinoma	LUAD	Lung squamous cell carcinoma	LUSC
Mesothelioma	MESO	Ovarian serous cystadenocarcinoma	OV	Pancreatic adenocarcinoma	PAAD
Pheochromocytoma and paraganglioma	PCPG	Prostate adenocarcinoma	PRAD	Rectum adenocarcinoma	READ
Sarcoma	SARC	Skin cutaneous melanoma	SKCM	Stomach adenocarcinoma	STAD
Testicular germ cell tumor	TGCT	Thyroid carcinoma	THCA	Thymoma	THYM
Uterine corpus endometrial carcinoma	UCEC	Uterine carcinosarcoma	UCS	Uveal melanoma	UVM

## Discussion

Smoking is a leading cause of preventable mortality worldwide. Smoking cessation can reduce the risk of disease, slow disease progression, and reduce mortality [22]. Clinician intervention increases the possibility that the patient will successfully quit smoking [23–25]. However, some studies have recently explored smoking-related epigenetic changes and identified some DNA methylation sites associated with smoking [26–28]. Additionally, smoking cessation may restore the DNA methylation status of former smokers to almost the level of never-smokers [13]. Little research is available for pairing DNA methylation data before and after short-term smoking cessation. Therefore, This research analyzed the level of DNA methylation in the whole blood of smokers in paired samples before and after successful short-term smoking cessation and explored the mechanisms underlying these associations to provide a basis for epigenetic changes after a short-term smoking cessation intervention.

Smoking alters DNA methylation. Smoking-associated DNA methylation can cause a variety of human diseases. The DNA methylation data analysis revealed that most DNA methylation levels were decreased after short-term smoking cessation, indicating that quitting smoking may reduce disease risk. In previous studies, the effects of smoking and smoking cessation on clinical characteristics have gained widespread attention. Studies have also demonstrated that smoking cessation is often accompanied by weight gain. However, the underlying mechanisms might include decreased metabolic rate, increased lipoprotein lipase activity, shift in food preference, and increased caloric intake [29]. In this current study, half of the eight individuals were prone to body weight gain after short-term cessation. Weight gain is also one of the reasons for the failure of smoking cessation. Weight gain can increase the risk of type 2 diabetes. Thus, taking interventions to prevent weight gain after quitting smoking is necessary. Chinn et al. showed that people who gave up smoking had a lower rate of lung function decline than those who continued to smoke [30]. The results of the present study showed that FEV1 and FVC tend to increase after quitting smoking; the differences are not significant; prompts to quit smoking can improve lung function decline to a certain extent. Smoking is a risk factor for asthma, and early smoke exposure is closely related to adolescent asthma [31, 32]. FeNO is a biomarker identifying allergic airway inflammation with a diagnostic value for asthma [33]. Although the duration of quitting smoking for these 8 individuals was only short-term, the level of FeNO was significantly decreased. Hence, encouraging people to give up smoking is necessary for asthma control.

The physiological mechanisms need to be better defined after quitting smoking; people also need to learn more about short-term smoking cessation. Bioinformatic analysis has provided an excellent way to explore the underlying cellular mechanisms. GO divides the function of genes into three parts: cellular component (CC), molecular function (MF), and biological process (BP). KEGG is a database of pathways. Genes corresponding to differential DNA methylation can be classified through GO terms and KEGG pathway enrichment analysis to explore the biological processes further. As GO analysis for genes corresponding to up- and downregulated DMCpGs, respectively, for genes corresponding to upregulated DMCpGs, the main enriched KEGG pathways include the AMPK signaling pathway and long-term depression signaling pathway. The AMPK and AMPK pathways are vital for energy metabolism, which promotes catabolic pathways to generate ATP. The involvement of AMPK may explain the changes in body mass after smoking cessation. Depression is one of the nicotine withdrawal syndromes [34]; also, the long-term depression pathway was highly enriched. A PPI network was used to display the association with biological signal transmission, gene expression regulation, energy and material metabolism, and cell cycle regulation. Next, the genes corresponding to up- and downregulated DMCpGs are used to construct the PPI network and identified the hub genes to identify the potential driver genes.

To increase the reliability, datasets correlated with smoking cessation in the NCBI GEO database were retrieved. After the systematic screening, GSE50660 was selected. The analysis of DMCpGs in these 8 patients’ data on differential DNA methylation levels between former and current smokers in GSE50660 revealed a total of 108 common methylation sites. GO/KEGG enrichment analysis was performed on the genes corresponding to these 108 methylation sites. KEGG enrichment analysis showed that the enriched of KEGG terms were cell cycle and human T-cell leukemia virus 1 infection. The abnormal cell cycle is the critical factor for tumorigenesis [35]. Many tumor therapies regulate cell cycle progression [36]; thus, the cell cycle is critical for tumor oncology. Smoking is an independent risk factor for many tumor types [37, 38] and may have deleterious effects on cancer treatment [39]. The 2014 Surgeon General’s report pointed out a causal correlation between smoking and all-cause and cancer-specific mortality and an increased risk of disease progression and tobacco-related second primary cancers. DNA methylation has been confirmed to be involved in tumor development and is also one of the characteristics of tumors [40]. Five genes were enriched in the cell cycle, including *CCNE1*, *CDC20*, *WEE1*, *YWHAZ*, and *MAD1L1*. The overexpression of *CCNE1* causes genetic instability of tumor cells and tumor-type development [41]. Multiple bioinformatics analyses suggested that *CDC20* could be a potential therapeutic target [42, 43]. Inhibition of *WEE1* might have a critical role in antitumor responses [44, 45]. *YWHAZ* promotes the progression of gastric cancer, prostate cancer, and hepatocellular carcinoma [46–48]. *MAD1L1*, also known as mitotic arrest deficient-like 1, was associated with a poor prognosis and insensitivity to paclitaxel in breast cancer [49]. The analysis showed that these five genes are highly expressed in most tumor types irrespective of paired or unpaired analysis using the TCGA database. Enriching genes related to the cell cycle after smoking cessation may be one of the reasons why smoking cessation decreases tumor-related mortality risk. Smoking is one of the main factors for lung cancer (50). Further explore the clinical implications of these five genes in lung cancer, providing indirect evidence of the advantages of smoking cessation.

Nevertheless, the present study has some limitations. All patients came from smoke cessation clinics. Patients who attended smoke cessation clinics were people who could not quit smoking by themselves. Despite the extensive efforts to promote smoking cessation, only some people take the initiative to quit smoking. Moreover, some disagreed with participating in the research, resulting in a small number of cases in this study. A total of 90 patients were recruited in this study. Unfortunately, only 13 people successfully quit smoking over the 3 − 6 months following enrolment. Eight of the 13 patients received fractional exhaled nitric oxide (FeNO) and lung function assessment before and after the target smoking cessation date. Therefore, all clinical data and DNA methylation analyses were based on these 8 patients. Influenced by the local characteristics, all the participants in the smoking cessation study were men, so this study lacks data on smoking cessation in women.

## Conclusions

DNA methylation sequencing was performed and a decrease in global DNA methylation in whole blood after short-term cessation for patients with smoking history. The clinical significance of this result was performed by the correlation analysis between CpG sites and clinical features. Furthermore, bioinformatics analysis was used to explore the potential mechanism. The changes indicate a general improvement in lung function gleaned from correlation analyses between CpG sites and clinical features. The data also confirms previous findings that short-term cessation can also lead to unwanted companion effects such as weight gain that may put patients at a higher risk of developing diabetes. This observation calls for monitoring patients that undergo short-term cessation, eventually using DNA methylation profiles for better surveillance.

## Data Availability

The datasets generated during the current study are available in the Gene Expression Omnibus (GEO) with the number GSE201532.
